# Whole‐brain radiotherapy plus sequential or simultaneous integrated boost for the treatment of a limited number of brain metastases in non‐small cell lung cancer: A single‐institution study

**DOI:** 10.1002/cam4.2696

**Published:** 2019-11-20

**Authors:** Dong Qing, Bin Zhao, Yi‐Chen Zhou, Hong‐Lei Zhu, Dai‐Yuan Ma

**Affiliations:** ^1^ Department of Oncology Affiliated Hospital of North Sichuan Medical College Nanchong Sichuan China

**Keywords:** brain metastases, neurocognitive, non‐small cell lung cancer, radiotherapy

## Abstract

**Background:**

To compare the survival outcomes and neurocognitive dysfunction in non‐small cell lung cancer (NSCLC) patients with brain metastases (BM ≤10) treated by whole‐brain radiotherapy (WBRT) with sequential integrated boost (SEB) or simultaneous integrated boost (SIB).

**Materials:**

Fifty‐two NSCLC patients with a limited number of BMs were retrospectively analyzed. Twenty cases received WBRT+SEB (WBRT: 3 Gy*10 fractions and BMs: 4 Gy*3 fractions; SEB group), and 32 cases received WBRT+SIB (WBRT: 3 Gy*10 fractions and BMs: 4 Gy*10 fractions; SIB group). The survival and mini‐mental state examination (MMSE) scores were compared between the groups.

**Results:**

The cumulative 1‐, 2‐, and 3‐year survival rates in the SEB vs SIB groups were 60.0% vs 47.8%, 41.1% vs 19.1%, and 27.4% vs 0%, respectively. The median survival times in the SEB and SIB groups were 15 and 10 months, respectively. The difference in survival rate was significant (*P* = .046). Subgroup analysis revealed that 1‐, 2‐, and 3‐year survival rates and median survival time in the SEB group were significantly superior to those of the SIB group, especially for male patients (age <60 years) with 1‐2 BMs (*P* < .05). The MMSE score of the SEB group at 3 months after radiation was higher than that of the SIB group (*P* < .05). Nevertheless, WBRT+SEB required a longer treatment time and greater cost (*P* < .005).

**Conclusions:**

WBRT + SEB results in better survival outcomes than WBRT+SIB, especially for male patients (age <60 years) with 1‐2 BMs. WBRT+SEB also appeared to induce less neurocognitive impairment than WBRT+SIB.

## Graphical abstract

Whole‐brain radiotherapy (WBRT)+sequential integrated boost (SEB) results in better survival outcomes than WBRT+simultaneous integrated boost (SIB), especially for male patients (age < 60 years) with 1‐2 brain metastases. WBRT+SEB also appeared to induce less neurocognitive impairment than WBRT+SIB.

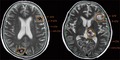

## 1 INTRODUCTION

Lung cancer is the leading cause of cancer‐related death and brain metastases (BMs), and up to 50% of patients develop central nervous system impairment, seriously affecting the survival outcome.^1^, ^2^, ^3^, ^4^ Non‐small cell lung cancer (NSCLC) accounts for the majority of lung cancer cases. Studies have shown that untreated NSCLC with BMs has a median survival of about 2 months.^5^ There are different treatment strategies for BMs, including surgery, stereotactic radiotherapy, whole‐brain radiotherapy (WBRT), targeted therapy, and immunotherapy.^6^, ^7^ Brown et al^7^ discussed mainstream methodologies for BMs. WBRT is the major treatment modality for unresectable BMs and for cases in which surgery and stereotactic radiosurgery (SRS) are not suitable.^8^ WBRT was first used to treat BMs in the mid‐1950s^9^ and was shown to increase the survival of patients to approximately 3 months.^10^ The QUARTZ trial suggested that WBRT provides limited benefit compared with best supportive care for poor‐prognosis NSCLC with asymptomatic BMs.^11^ However, for patients with clinical symptoms of BMs, WBRT can also alleviate the neurological symptoms and improve local control of the tumor.^12^ Approximately two‐thirds of patients who receive WBRT are able to receive a reduced corticosteroid dose upon alleviation of brain symptoms, which supports the use of WBRT as a palliative treatment.^13^ Several prospective studies have demonstrated that WBRT combined with lesion‐targeting radiotherapy boost is associated with better overall survival (OS) and a better local control rate when the number of BMs is small,^12^, ^14^, ^15^ especially in patients with favorable prognosis, whereas hypofractionated stereotactic radiotherapy may be effective for larger and more BMs.^16^, ^17^, ^18^ At present, there are two main boost schemes: sequential integrated boost (SEB), in which the boost dose is delivered after WBRT, and simultaneous integrated boost (SIB), in which the boost dose is delivered within a fraction but varied throughout the course the treatment.^19^, ^20^, ^21^, ^22^ There are no definitive regimens for integrating WBRT with local boost for BMs. With the advancement of comprehensive treatment of tumors, such as targeted therapy and immunotherapy, the survival time of patients with BMs has been prolonged. However, neurocognitive impairment caused by radiation brain injury is becoming more and more prominent, which seriously affects the quality of life of patients. The aim of this study was to analyze the clinical efficacy of WBRT combined with SEB or SIB in NSCLC cases with a limited number of BMs and to compare the resultant neurocognitive impairment between schemes. This study was a single institutional retrospective analysis.

## 2 METHODS

### 2.1 Clinical information

This retrospective study was approved by the Review Board of the affiliated Hospital of North Sichuan Medical College (No. 2017ER(A)007). Fifty‐two NSCLC patients with 10 or fewer BMs were included between January 2013 and December 2016. Informed consent was collected from all patients.

The patient eligibility criteria for this study were as follows: (a) histological confirmation of NSCLC and contrast‐enhanced computed tomography (CT) or magnetic resonance imaging (MRI) confirmation of ≤10 intracranial lesions of metastases before treatment; (b) completed treatment with corresponding follow‐up information; (c) Karnofsky Performance Status score ≥60; (d) expected survival time ≥1 month; and (e) maximum diameter of BMs ≤5 cm.

The patient exclusion criteria for this study were as follows: (a) BM close (within 5 mm) to brainstem or optic apparatus; (b) leptomeningeal metastases according to cytological or imaging evidence; (c) negative targeted drug‐related gene test; (d) incomplete mini‐mental state examination (MMSE) score; (e) history of surgical treatment or cranial RT; and (f) any contraindications to contrast CT/MRI.

### 2.2 Patient characteristics and manifestations

**Figure 1:**
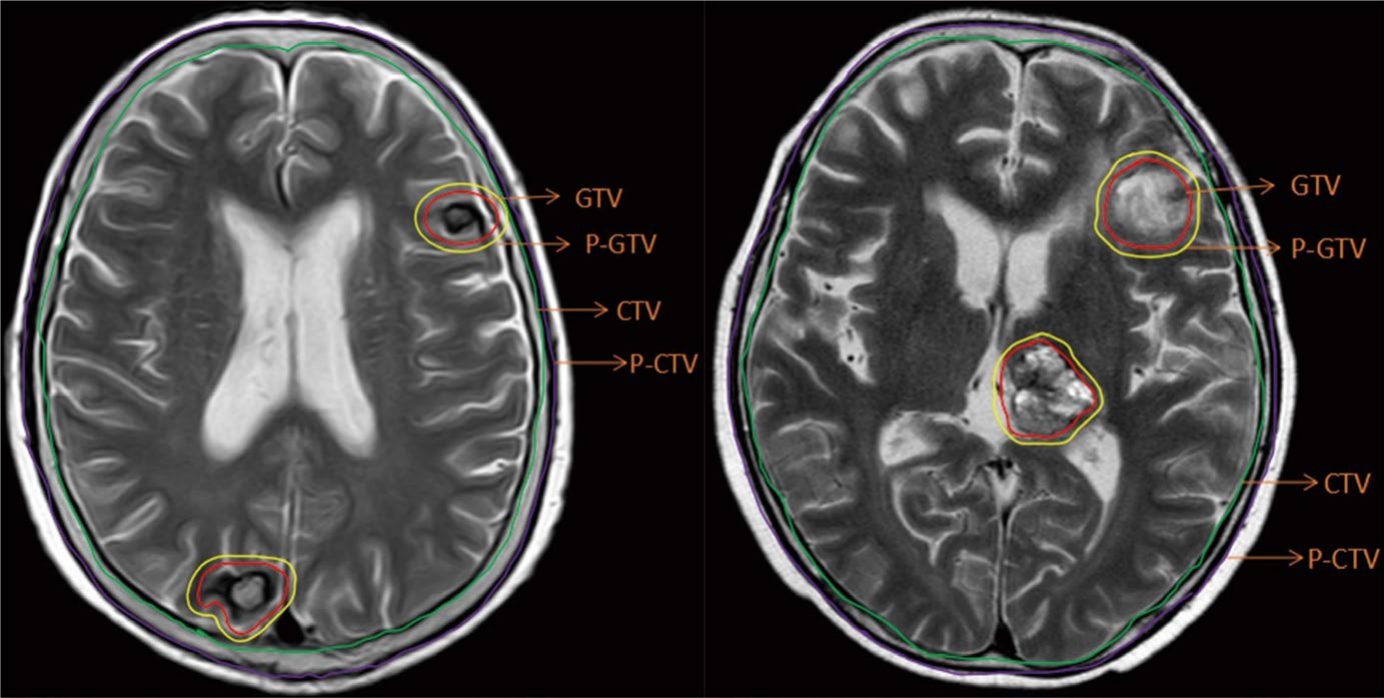
Delineation of important RT volumes (GTV, CTV, PTV, etc) on T2‐weighted MRI. CTV, clinical target volume; GTV, gross tumor volume; MRI, magnetic resonance imaging; PTV, planning target volume

The study population included 52 patients with a median age of 59 years. The SEB group included 20 patients (14 males), of whom 11 patients had no <3 BMs. The SIB group included 32 patients (22 males), of whom 17 cases had no <3 BMs. The detailed patient characteristics are shown in Figure 1 and Table 1. Almost all patients received chemotherapy and some received lung radiotherapy. Chemotherapy regimens are the first choice recommended by the NCCN guidelines. The dose of gross tumor volume (GTV) for radiotherapy was 66 Gy/33 fractions. There was no statistical difference between the two groups.

**Table 1:** Patient characteristics

Characteristic, n	SEB group	SIB group	χ^2^/t	*P*
Gender				
Male	14	22	0.09	1.000
Female	6	10		
Age (y)				
<60 y old	6	8	0.156	.754
≥60 y old	14	24		
Number of lesions				
1‐2	9	15	0.017	1.000
≥3	11	17		
KPS score				
≥70	16	22	0.792	.524
<70	4	10		
Pathology				
Adenocarcinoma	11	20	0.693	.553
Non‐adenocarcinoma	9	12		
Clinical stage				
IVa	7	11	0.002	1.000
IVb	13	21		

Note

The SEB group received WBRT + SEB; the SIB group received WBRT + SIB.

Abbreviations: KPS, Karnofsky Performance Status; SEB, sequential integrated boost; SIB, simultaneous integrated boost; WBRT, whole brain radiotherapy.

All patients completed RT without radiation‐induced death. Almost all of the patients suffered from acute craniocerebral injury and edema. Dizziness and headache were treated with routine mannitol and methylprednisolone after treatment.

### 2.3 Treatment planning and delivery

**Figure 2:**
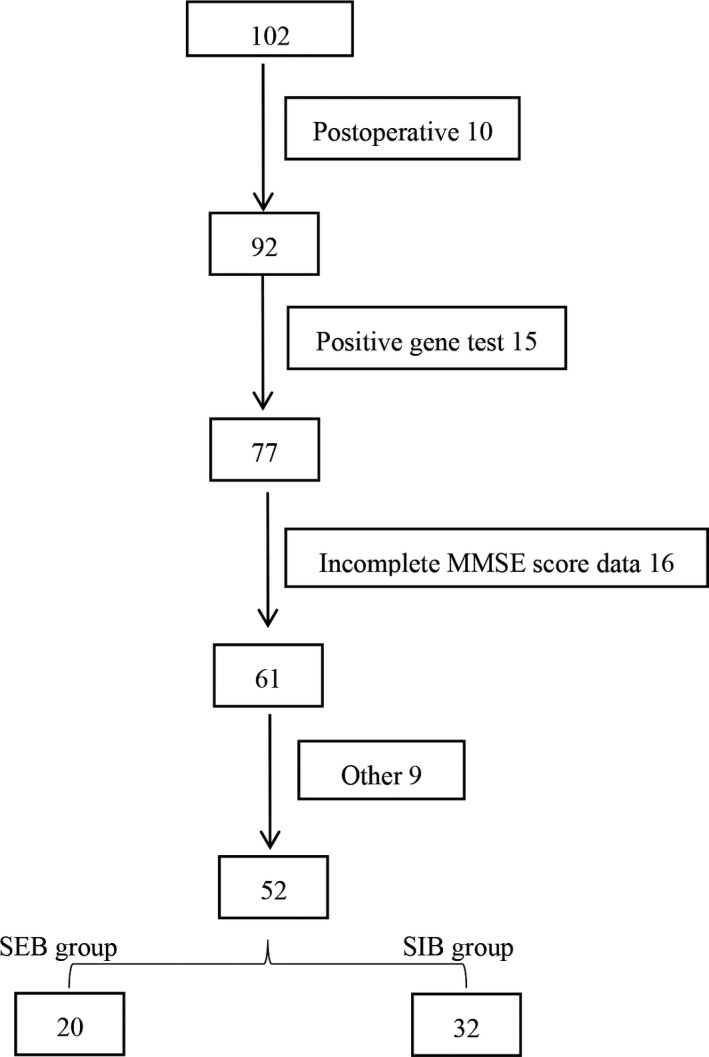
Patient inclusion flow chart

Patients were divided into two groups according to the treatment scheme. The SEB group received WBRT at a dose of 30 Gy/10 fractions (5 fractions per week) with SEB on BMs of 12 Gy/3 fractions. The SIB group received WBRT at a dose of 30 Gy/10 fractions (5 fractions per week) with SIB of 40 Gy/10 fractions. All treatments were delivered using intensity‐modulated RT (IMRT). The biological effective dose (BED) was calculated based on a linear‐quadratic model (BED = nd [1 + *d*/(*α*/*β*)], *α*/*β* = 10 Gy). The BED for the whole brain and metastases in the SEB group were 39 and 55.8 Gy, respectively, and those in the SIB group were 39 and 56 Gy, respectively. The clinical target volume (CTV) for WBRT was the entire brain. The planning target volume was an isotropic expansion with a margin of 5 mm to the CTV. The GTV of lesion was delineated based on contrast enhancement on MRI. The planning GTV was calculated by adding a 3D isotropic margin of 2 mm to the GTV (Figure 2).

### 2.4 Neurocognitive assessment, survival, and follow‐up

All patients received the MMSE before treatment, at the end of treatment, and 1, 3 and 6 months after treatment for assessment of neurocognitive function. OS was defined as the duration from the start of RT to the last day of follow‐up or death. Follow‐ups were performed by telephone every month for all patients.

### 2.5 Statistical analysis

All statistical analyses were performed using SPSS 22.0 software. The Kaplan‐Meier method was used to analyze the survival rates and median survival times. The survival rates were compared between the groups using the Log‐rank method. Student's *t* test was applied to compare overall treatment times and MMSE scores. Other quantities were compared by either chi‐square (*χ* ^2^) or Fisher's exact test. *P* < .05 was considered statistically significant.

## 3 RESULTS

### 3.1 WBRT+SEB was associated with superior survival outcomes compared with WBRT+SIB

**Figure 3:**
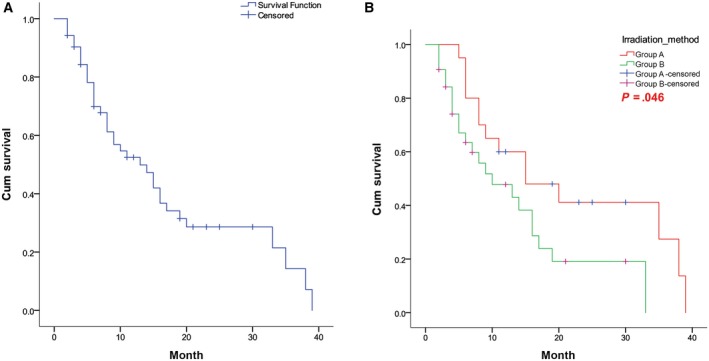
Survival curves of (A) all patients and (B) patients in the SEB and SIB groups. SEB, sequential integrated boost; SIB, simultaneous integrated boost

Among the 52 patients, the cumulative 1‐, 2‐, and 3‐year survival rates were 52.5%, 28.6%, and 14.3%, respectively, and the median survival time was 13 months (Figure 3A). With the different boost schemes, the cumulative 1‐, 2‐, and 3‐year survival rates in the SEB vs SIB groups were 60.0% vs 47.8%, 41.1% vs 19.1%, and 27.4% vs 0.0%, respectively. The median survival times of the SEB and SIB groups were 15 and 10 months, respectively. The difference in survival rate was significant (*P* = .046; Figure 3B).

### 3.2 WBRT+SEB was associated with improved survival in patients with 1‐2 BMs

**Figure 4:**
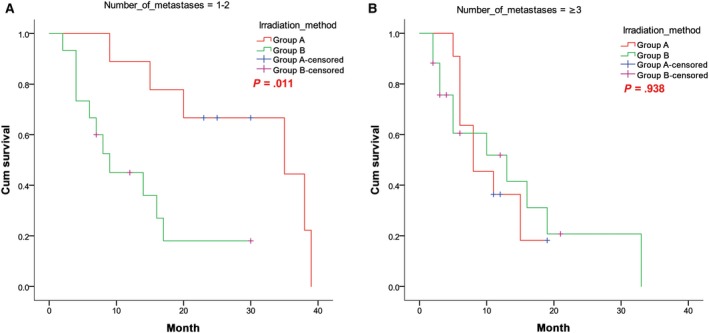
Survival curves of patients with (A) 1‐2 BM(s) and (B) ≥3 BMs in both groups. BM, brain metastases

For 24 patients with 1‐2 BM(s), the cumulative 1‐, 2‐, and 3‐year survival rates for the SEB group (n = 9) vs SIB group (n = 15) were 88.9% vs 45.0%, 66.7% vs 18.0%, and 44.4% vs 0.0%, respectively. The median survival times of these subgroups within the SEB and SIB groups were 35 and 9 months, respectively. The survival of these patients in the SEB group () was significantly better than that of this subgroup within the SIB group (*P* = .011). However, the survival of patients with ≥3 BMs (28 patients in both groups) did not differ significantly between the SEB and SIB groups (*P* = .938). The corresponding survival curves are shown in Figure 4A,B.

### 3.3 WBRT+SEB was associated with improved survival in male patients

**Figure 5:**
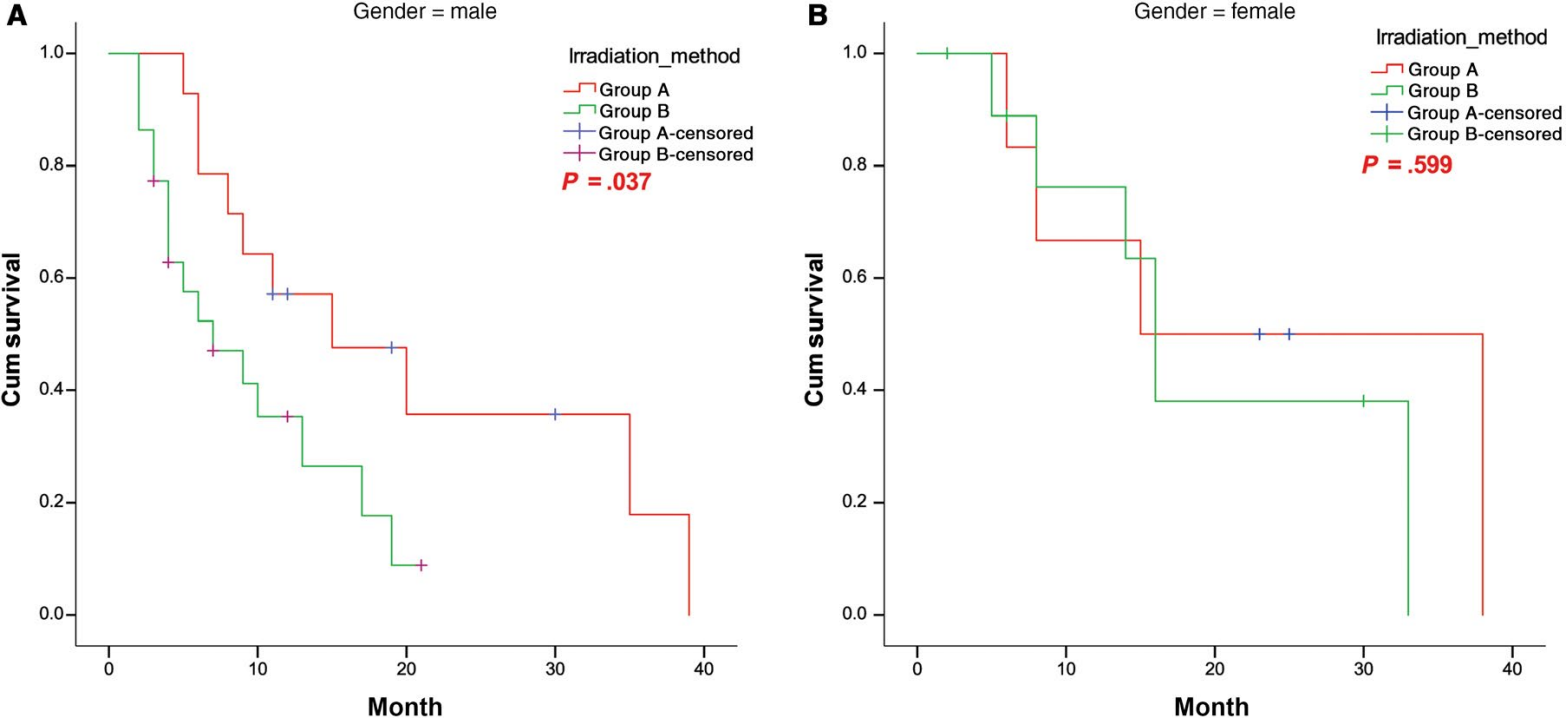
Survival curves of (A) male and (B) female patients in the SEB and SIB groups. SEB, sequential integrated boost; SIB, simultaneous integrated boost

The cumulative 1‐, 2‐, and 3‐year survival rates of male patients (n = 36) in the SEB group (n = 14) vs the SIB group (n = 22) were 57.1% vs 35.3%, 35.7% vs 8.8%, and 17.9% vs 0.0%, respectively. The median survival times of male patients in the SEB and SIB groups were 15 and 7 months, respectively. The survival of male patients in the SEB group was significantly better than that of male patients in the SIB group (*P* = .037). The survival curves for male patients in both groups are shown in Figure 5A.

In contrast to the results for female patients, no significant difference in survival between the groups was observed for female patients (*P* = .599). The survival curves for female patients in both groups are shown in Figure 5B.

### 3.4 WBRT+SEB was associated with improved survival in patients <60 years of age

**Figure 6:**
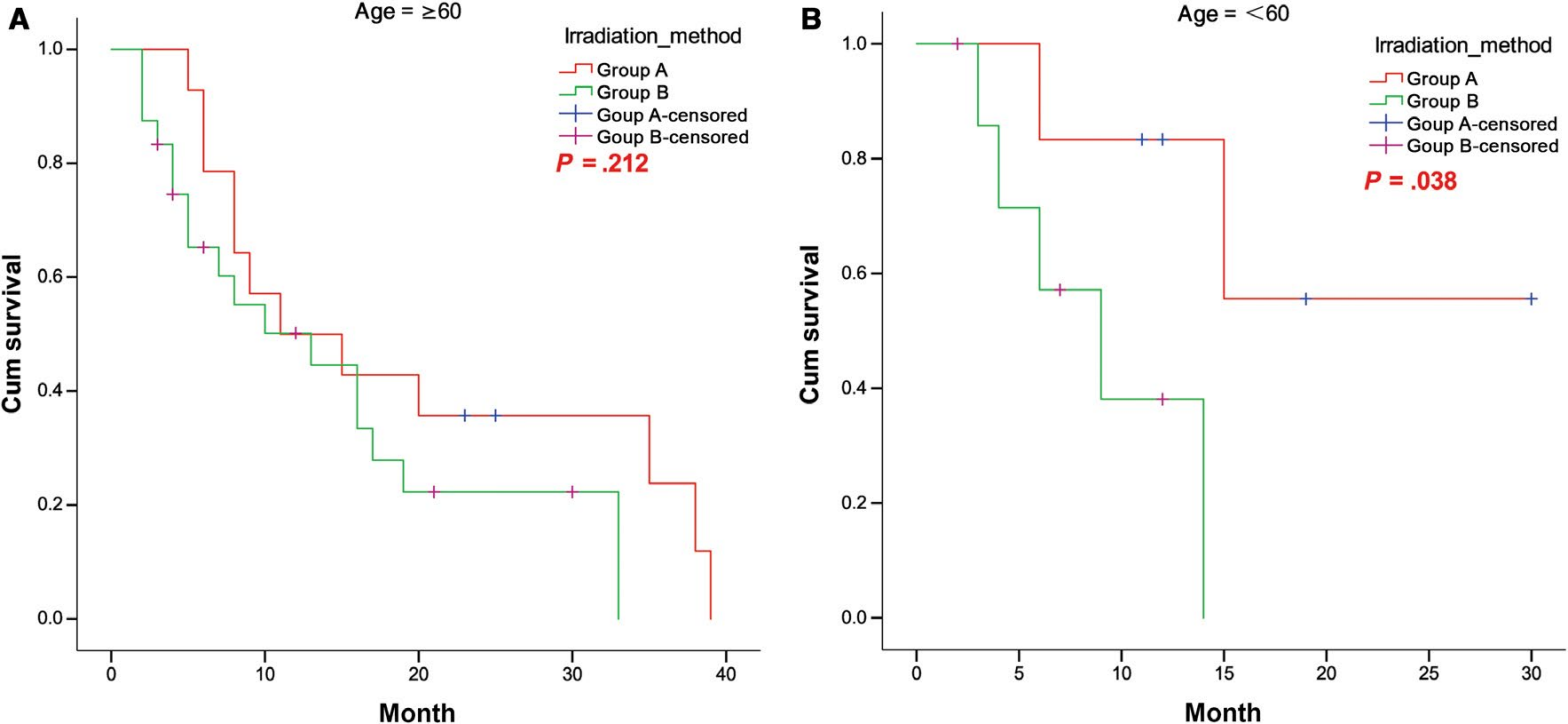
Survival curves of patients with (A) age ≥60 years and (B) age <60 years in the SEB and SIB groups. SEB, sequential integrated boost; SIB, simultaneous integrated boost

The cumulative 1‐, 2‐, and 3‐year survival rates of patients ≥60 years old in the SEB group (n = 14) vs the SIB group (n = 24) were 50.0% vs 50.2%, 35.7% vs 22.3%, and 23.8% vs 0%, respectively. No significant difference in survival of patients ≥60 years was found between the groups (*P* = .212). For patients <60 years, the cumulative 1‐, 2‐, and 3‐year survival rates in the SEB group (n = 6) vs the SIB group (n = 8) were 83.3% vs 38.1%, 55.6% vs 0.0%, and 0.0% vs 0.0%, respectively. The difference in the survival of patients <60 years between the two groups was significant (*χ* ^2^ = 4.31, *P* = .038). The corresponding survival curves are shown in Figure 6A,B.

### 3.5 WBRT+SEB resulted in less severe cognitive impairment at 3 months after treatment

The MMSE scores of the SEB and SIB groups before treatment, at the end of treatment, and 1, 3, and 6 months after treatment were tabulated (Table 2). For patients in the SEB group, the scores at the end of treatment (24.90 ± 2.150) and at 1 month after treatment (26.30 ± 1.838) did not differ from the baseline score (*P* > .05). However, the MMSE scores at 3 months (24.90 ± 1.410) and 6 months (23.58 ± 2.545) after treatment were lower than the baseline score (*P* < .05). Similarly, in the SIB group, the MMSE scores at the end of treatment (24.72 ± 2.174) and 1 month after treatment (26.06 ± 1.366) did not differ from the baseline score. However, the MMSE scores at 3 months (23.39 ± 1.853) and 6 months (23.14 ± 2.971) after treatment were lower than the baseline score (*P* < .05).

**Table 2:** MMSE scores before and after treatment in the SEB and SIB groups

	SEB group	SIB group
Pre‐radiation (baseline*)	25.95 ± 2.282	25.06 ± 2.940
At the end of radiation	24.90 ± 2.150	24.72 ± 2.174
*P* vs baseline*	.108	.580
Baseline_1_	25.95 ± 2.282	25.06 ± 2.940
1 mo after radiation	26.30 ± 1.838	26.06 ± 1.366
*P* vs baseline_1_	.580	.065
Baseline_3_	25.95 ± 2.282	25.04 ± 3.097
3 mo after radiation	24.90 ± 1.410	23.39 ± 1.853
*P* vs baseline_3_	**.031**	**<.001**
Baseline_6_	25.79 ± 2.226	25.19 ± 3.400
6 mo after radiation	23.58 ± 2.545	23.14 ± 2.971
*P* vs baseline_6_	**<.001**	**<.001**

Note

Bold value indicates statistical differences.

Abbreviations: MMSE, mini‐mental state examination; SEB, sequential integrated boost; SIB, simultaneous integrated boost.

MMSE scores were stratified according to group, number of lesions, and treatment time (Table 3). The MMSE score at 3 months after treatment for patients with 1‐2 BM(s) in the SEB group (25.00 ± 1.225) was higher than that of these patients in the SIB group (23.50 ± 1.732; t = 2.210, *P* = .040). The MMSE score at 3 months after treatment for patients with 3 or more BMs in the SEB group (24.82 ± 1.601) was significantly higher than that of these patients in the SIB group (23.31 ± 1.991; t = 2.084, *P* = .048).

**Table 3:** Subgroup analysis of MMSE scores with different number of BMs

Timing	SEB group	SIB group	*P*
	1‐2 lesions		
Pre‐radiation	26.11 ± 2.028	25.00 ± 3.719	.426
At the end of radiation	24.89 ± 2.619	24.54 ± 1.984	.724
1 mo after radiation	26.44 ± 1.509	26.08 ± 0.954	.491
3 mo after radiation	25.00 ± 1.225	23.50 ± 1.732	**.040**
6 mo after radiation	23.78 ± 2.224	23.00 ± 3.808	.604
	≥3 lesions		
Pre‐radiation	25.82 ± 2.562	25.11 ± 2.378	.448
At the end of radiation	24.91 ± 1.814	24.84 ± 2.340	.936
1 mo after radiation	26.18 ± 2.136	26.05 ± 1.615	.853
3 mo after radiation	24.82 ± 1.601	23.31 ± 1.991	**.048**
6 mo after radiation	23.40 ± 2.914	23.25 ± 2.340	.895

Note

Bold value indicates statistical differences.

Abbreviations: BMs, brain metastases; MMSE, mini‐mental state examination; SEB, sequential integrated boost; SIB, simultaneous integrated boost.

### 3.6 WBRT+SEB required a longer treatment time and greater cost compared with WBRT+SIB

Patients in the SEB group completed treatment within an average time of 16.16 ± 2.21 days, whereas patients in the SIB group completed treatment within an average time of 13.25 ± 1.82 days. SEB group treatment takes longer than SIB group treatment and costs about $1000 more. The difference was statistically significant (*P* < .001).

## 4 DISCUSSION

The current study compared the survival outcomes of WBRT combined with sequentially (SEB group) or simultaneously (SIB group) integrated boost for the treatment of a limited number of BMs in NSCLC patients. A comparative analysis showed that the cumulative 1‐, 2‐, and 3‐year OS rates or median survival times in the SEB group were better than those in the SIB group. Subgroup analyses indicated that male patients, patients with 1‐2 BM(s), and patients <60 years old who received WBRT+SEB had better survival outcomes. The results of this study are basically consistent with those of Dobi et al,^23^ who reported 468 patients with BMs from various primary tumors who were treated with 10 fractions of 3 Gy WBRT, WBRT plus 10 fractions with 2 Gy boost, or WBRT with simultaneous boost in 15 fractions of 2.2 Gy WBRT plus 0.7 Gy boost. They found that OS was better with whole‐brain irradiation with integrated boost, and SEB was associated with better survival than SIB.^23^ The results of our current study suggest that WBRT combined with SEB leads to better survival outcomes. First, this might be because cancer cells that remain after WBRT experience hypoxia, and SEB provided a sufficient time for re‐oxygenation, thus increasing the radiosensitivity of cancer cells. Second, WBRT led to a tumor volume reduction and cerebral edema, and contouring of the GTV for SEB improved the degree of tumor overlap with the target area. Lastly, SEB was associated with less damage to normal brain tissue than SIB. Although WBRT provides survival benefits to patients, the radiation brain injury cannot be ignored. Ebi et al^24^ reported that the incidence of leukoencephalopathy was 34.4% at >6 months after WBRT. Vigliani et al^25^ reported that about 2%‐5% of long‐term survivors who received fractionated WBRT had cognitive impairment including memory loss, ataxia, and urinary incontinence. Nieder et al^26^ showed that 49% of patients experienced significant neurocognitive impairment at 2 years after WBRT with conventional fractionation. Robbins et al^27^ reported that changes in cognitive function may occur even in primatized brains upon exposure to 10 Gy. Li et al^8^ reported similar neurocognitive dysfunction, which may be related to hippocampal damage.

The MMSE is a commonly used to assess neurocognitive function in patients with BMs.^28^ The MMSE is highly reproducible and reliable, and it is also sensitive and specific for the diagnosis of dementia.^29^ The MMSE evaluates short‐term memory, language proficiency, computational skills, use and attention, orientation, and other aspects. Previous research has demonstrated that the MMSE score is associated with survival in patients with BMs.^30^ In this study, the MMSE scores at the end of treatment and at 1 month after treatment did not differ from baseline scores, whereas the MMSE scores at 3 and 6 months after treatment were significantly lower than baseline scores (*P* < .05). These data indicated that radiation‐induced neurocognitive dysfunction occurred 3 months after WBRT, which is consistent with previous findings. Gondi et al^31^ reported that 30% of patients experience a decline in memory function 4 or 6 months after whole‐brain irradiation. Similarly, Slotman et al^32^ reported that the neurocognitive decline was most obvious at 3 months after preventive WBRT in 286 patients with extensive SCLC. Brown et al^33^ reported that cognitive impairment at 3 months was observed more frequently after WBRT+SRS compared to SRS alone. There was more deterioration in the arm in immediate recall, delayed recall and verbal fluency for WBRT+SRS. After WBRT+SRS, there was more deterioration in overall QOL (*P* = .001) and functional well‐being (*P* = .006) at 3 months. WBRT offers a higher control rate of intracranial metastases compared with SRS, but also leads to more serious cognitive impairment. However, study showed that Tomotherapy can better protect the hippocampus and reduce the radiation dose of the hippocampus for WBRT.^34^ The use of Tomotherapy for WBRT may reduce the neurocognitive decline caused by radiation brain injury. This is basically consistent with the results of our study. In current study, the MMSE scores at 3 and 6 months after treatment in SEB group were higher than those of the SIB group, suggestive of a greater extent of neurocognitive impairment upon treatment with WBRT+SIB.

## 5 CONCLUSIONS

In summary, WBRT+SEB was associated with better survival outcomes for NSCLC patients with a limited number of BMs than was WBRT+SIB, especially in male patients, patients aged <60 years, and patients with only 1‐2 metastases. A decline in neurocognitive function occurred 3 months after treatment with both boosting schemes, but patients who received WBRT+SEB showed less impairment. Notably, the number of cases in this study was small, and a retrospective analysis may introduce potential bias in the screening of cases. Lastly, the time over which neurocognitive impairment was assessed after WBRT was relatively short. Future prospective studies are needed to reveal to better understand the cognitive impairment in greater detail.

## CONFLICT OF INTEREST

The authors declare that they have no conflict of interests.

## Data Availability

The datasets used and/or analysed during the current study are available from the corresponding author on reasonable request.
